# Treatment of Patients with Low Back Pain: A Comparison of Physical Therapy and Chiropractic Manipulation

**DOI:** 10.3390/healthcare8010044

**Published:** 2020-02-24

**Authors:** Nima Khodakarami

**Affiliations:** Department of Health Policy and Management, Texas A&M University, College Station, TX 77843, USA; nima@tamu.edu

**Keywords:** chiropractic, physical therapy, treatment outcome, low back pain, therapy, economics, patient satisfaction, recurrence, health care costs, illness

## Abstract

Low back pain (LBP) is a pandemic and costly musculoskeletal condition in the United States (U.S.). Patients with LBP may endure surgery, injections, and expensive visits to emergency departments. Some suggest that using physical therapy (PT) or chiropractic in the earlier stage of LBP reduces the utilization of expensive health services and lowers the treatment costs. Given that there are costs and benefits with each of these treatments, the remaining question is in a short period of time which of these treatments is optimal. The purpose of this study was to investigate the cost-effectiveness of chiropractic versus PT in the U.S. A decision tree analytic model was used for estimating the economic outcomes. The findings showed that the total average cost in the chiropractic group was $48.56 lower than the PT group. The findings also showed that the daily adjusted life years (DALY) in the chiropractic group was 0.0043 higher than the PT group. Chiropractic care was shown to be a cost-effective alternative compared with PT for adults with at least three weeks of LBP over six months.

## 1. Introduction

Low back pain (LBP) is a chief cause of years lost to disability in the world [1]. It is also an encumbrance to patients and a burden to societies. In industrialized countries, LBP causes a high cost of medical expenses and loss-of-work [2,3,4]. Only in the U.S., over $80 billion are directly and indirectly spent in the treatment of LBP, of which $7.4 to $28 billion constitute the cost of loss-of-work, and $26 billion constitute the cost related to pharmacologic, non-pharmacologic, and therapies. In fact, LBP is the second most common reason for visits to physicians in the US [5,6]. 

While LBP commonly resolves within 8 to 12 weeks, it can persist beyond 3 months in 15% of patients, where it becomes a chronic pain [5,7]. Thus, it is essential to identify and promote early interventions for acute LBP that are cost-effective [8,9]. Such interventions are necessary as the burden of chronic back pain may lead to a lower productivity, lower quality of life, and higher financial liability for society [10]. 

To manage acute LBP, clinical guidelines recommend physical activity, the use of pharmacologic therapies, and less use of routine imaging. The reason for less emphasis on imaging is that exposure to radiation can raise the risk of carcinogenesis and teratogenesis, which induce unregulated growth processes in cells or tissues. It also imposes dollar charges to the patients. However, it can lower the days of suffering from undiagnosed disease (e.g., infections of spine or bone tumor) during the period of treatment [11,12]. In terms of pharmacologic therapies, the use of acetaminophen or nonsteroidal anti-inflammatory drugs, in conjunction with self-care are suggested. However, if these types of care management do not heal patients, non-pharmacologic therapies including PT and chiropractic therapy are recommended [13].

PT, for example, is shown to have better outcomes than medical treatment or no treatment at all [14]. A study of the cost-benefits of an early treatment of acute LBP with PT has shown that the outcome for PT is superior to no-treatment or medical intervention alone at one-year follow-up [9]. A similar study found that, at one-year follow-up, physical therapy compared with the usual primary care is a cost-effective treatment for patients with acute and nonspecific LBP [15].

Also, it is shown that PT reduces the utilization of specific types of care (e.g., diagnostic imaging procedures, magnetic resonance imaging (MRI), use of injection procedures, use of fluoroscopically-guided procedures, and prescription medication). Accordingly, receiving PT reduces the likelihood of incurring high charges for subsequent healthcare [16]. 

Unlike PT, the assessment of patients in chiropractic treatment depends on imaging (e.g., X-rays, computed tomography (CT), and MRI) [17]. Nevertheless, chiropractic has shown to have similar benefits to PT. Carey et al. (1995) found that those patients who are treated by chiropractors are more satisfied than those who are treated by orthopedic surgeons [8]. Goertz et al. (2013) also found statistically and clinically significant benefits to those who receive additional chiropractic manipulative therapy compared with those who only receive standard medical care [18]. Hurwitz et al. (2002) showed that the effectiveness of chiropractic care is similar to medical care for LBP after 6 months of follow-up. It is also shown that PT is marginally more effective than medical care alone for reducing disability in some patients [19]. 

Thus, both chiropractic therapy and PT, compared with other non-pharmacologic interventions, are shown to have better, yet similar benefits, and at lower costs [7,20]. Overall, it is shown that PT and chiropractic manipulation have similar effects and costs for patients with LBP [17].

While the assessment of acute LBP focusing on the cost-effectiveness of PT and chiropractic within a short period of time in the U.S is rare [8,21,22], the remaining question is which one of these two treatments is optimal. This paper aims to examine the posed question while it attempts to fill the gap by studying the cost-effectiveness of chiropractic compared with PT within 6 months of treatment and follow-up.

## 2. Materials and Methods 

A decision tree model was used for the cost-effectiveness analysis. Model inputs were extracted from the existing literature. The probabilities of certain results and the final outcomes of a decision path were adjusted according to the national estimates. For data collection, Medline and PubMed databases were searched from 2000 to September 2018. In search of PubMed, ten clinical studies that examined the costs and outcomes of non-pharmacologic treatments for LBP were found. Studies were chosen for a further detailed analysis based on patients’ country of residence, types of treatment, duration of treatment, and types of back pain (acute vs. chronic). Any studies that did not describe the source of participants and the methods of sampling were excluded. Additionally, those studies that did not have any records of patients’ return to work, patients’ disability, or their duration of LBP were excluded. Accordingly, two articles that focused on the foreign countries were excluded from the pool of studies. From the remaining studies, only two articles have examined the cost-effectiveness of either PT or chiropractic in the US. The MedLine (Ovid) was also searched for studies that examined non-pharmacologic treatments for LBP. Eleven articles were found, of which four studies that examined the cost-effectiveness of chiropractic and physiotherapy versus the placebo test were included for further analysis. 

After selecting the studies, data was extracted as follows. First, patient-level data from prior studies was collected by their treatment group. Second, the duration of care and probability of patients being cured after each stage of treatment were collected for both treatment groups. In each group, patients were stratified by their gender and their observable characteristics (e.g., age, smoker, history of LBP, sick-leave days, etc.). Third, the weighted mean resource use and costs for each stratum were estimated. To evaluate the characteristics for an even spread across the groups, a t-test and chi-square test were used. The cost-effective estimation was drawn from the estimated sample. 

### 2.1. Sample of Study

The patient’s sample included both men and women aged to 18 to 60 years old with a mean age of 40 years. All samples had at least three weeks of LBP over six months and experienced one of the two non-pharmacologic treatments, including PT and chiropractic. Fifty-five percent of the population were assigned to the chiropractic group, and the remaining 45% were assigned to the PT treatment. The pregnant patients or those who previously received treatment for back pain were excluded from the selected sample. The summary of patients’ characteristics is depicted in Table 1.

To assess the outcome in the short-term, this article focuses on the effects of the assigned treatment on sick-leave days [23]. A high duration of sick leave may halt employee engagement and impose economic costs on the organization [24,25]. Correspondingly, this study uses the duration of sick leave before treatment for chiropractic and physiotherapy groups. The literature showed that in the chiropractic group, 61% of the individuals were on one or less than a week of sick leave, and 37% of the individuals were on one to four weeks of sick leave. For the physiotherapy group, 58% of the individuals were on one or less than a week of sick leave, 33% of the individuals were on one to four weeks of sick leave, and only 9% of the individuals reported one month of sick leave. Immediately after chiropractic treatment, with 4.9 sessions over the four weeks during which treatment occurred, the sick-leave days dropped by 40%. Similarly, after PT, with 6.4 sessions over the four weeks during which treatment occurred, the sick-leave days dropped by 43% [3,21]. 

After the first month of treatment, people in the chiropractic and PT groups sought further sessions of chiropractic or PT. Noting this, there has been an additional 5.2 percent of visits to physical therapy by the chiropractic group and 6.7 percent of visits to chiropractic by the PT group. Accordingly, the overall number of visits within six months for the PT group was 20 percent higher than for the chiropractic group. After the six-months follow-up, the number of sick leave days for the chiropractic and PT group dropped by 48 percent and 46 percent, respectively [3,21].

### 2.2. Costs of Treatments

Primarily, all costs were obtained for 1995 and adjusted for 2018. Given the mean costs of treatment in 1995 dollars, this paper spotted the cost of the visits, the imaging cost for each treatment group, along with the copayments for drugs and visits, and the additional costs including laboratory tests and medications to the patients. The imaging strategy was shown to have the benefits of reducing days of suffering from undiagnosed disease by 0.04 days compared with no imaging, which left the patient with the pain of undiagnosed and untreated disease. However, it imposed the risk of irradiation (16.81 mrad) with additional imaging charges. Accordingly, to avoid one day of suffering, it was estimated that radiation imposed a risk of 3188 mrad with a $2072 cost [26]. 

With a three-percent rate of inflation, costs were updated to the 2018 dollar values. Furthermore, the updated dollar values of visits were cross-checked with the more recent payments to ensure they did not surpass the cap payments for the utilization of treatments [27]. Thus, the proposed costs were recorded in Table 2.

### 2.3. Statistical Analysis

**Figure 1:**
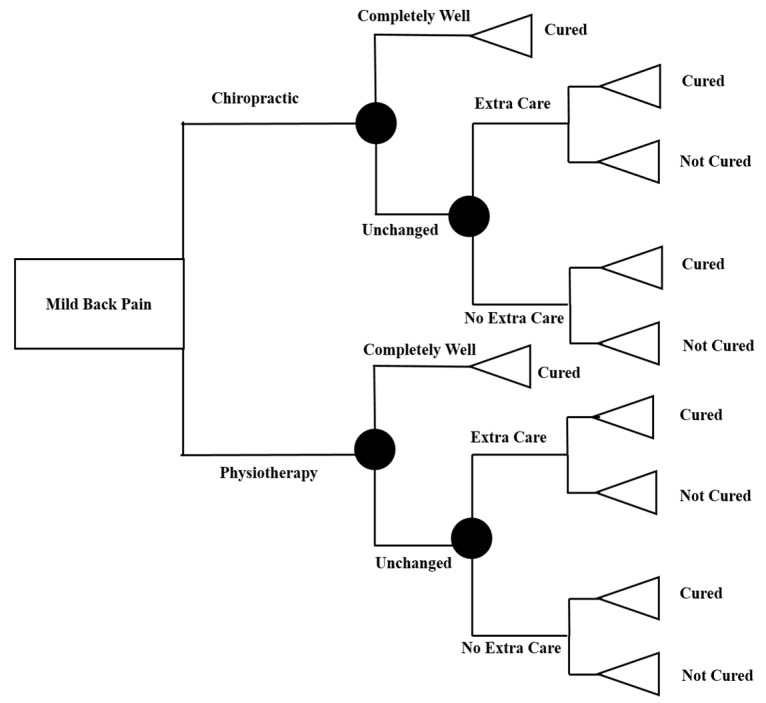
Decision tree.

According to the literature, 66% of patients who went to chiropractic groups experienced complete wellness right after treatment. From this, it was assumed that 34 percent of patients should consume additional healthcare after treatment by up to six months. In terms of recurrence at six months, 21% were shown to feel pain. Among the physiotherapy group, the literature showed that 56% of the patients experienced complete wellness right after treatment. Thus, it was assumed that 44% should have additional health care utilization of up to six months after the treatment period [3,21]. Given these assumptions, the decision tree was drawn below (*See* Figure 1). The cost and disability-adjusted life-years (DALY) was estimated at each node. To estimate DALY, the sick-leave days was used as the measure of disability average life years. To estimate DALY, the following formula was used:

(1) $$\mathrm{DALY}\;\mathrm{gained}=(D_{i}-D)*\left(1-e^{-rl}\right)/r$$

where D is a disability, L is the duration of pain, and r is a discount rate, equaling 3%. For the duration of pain, one month and six months were used for the DALY estimation. 

## 3. Results

The findings showed that for the six months of treatment, the mean cost of chiropractic was 410.89. The findings also showed that the mean cost of physiotherapy was 459.45 for the same treatment duration. In the estimation process, the cost-sharing of patients e.g., copayments for visits were ignored. Therefore, the items that were incorporated in this estimation include both direct and indirect costs that were covered by the insurance. It was also assumed that the employer was covering all the costs of insurance and carried the costs of the sick leave. Furthermore, other indirect costs, including costs to the relatives and family, transportation, loss of a job, and change of job, were excluded from the estimation. The summary of the results is illustrated in Table 3.

**Figure 2:**
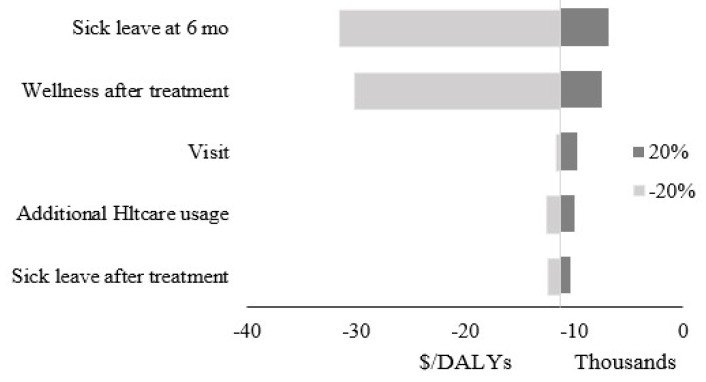
Tornado graph.

Given these findings, chiropractic compared with PT is the cost-effective choice, with savings of $48.56 and an increase in DALY of 0.004. The sensitivity analysis of this study was conducted based on a 20% increase and decrease in the fraction of people that felt well after treatment, the use of additional treatment of chiropractic/PT, changes in the number of their sick leave days after treatment and at six months, and the number of visits. The tornado graph is depicted in Figure 2.

The sensitivity analysis for this study showed that the incremental cost-effectiveness ratio was robust to the varying assumptions. The tornado graph showed the outcomes of changes in the assumptions within a 20% range. The most considerable variation in the outcome are obtained from changes in the ratio of the sick leave after six months from the beginning of treatment, followed by the percentage of people who reported wellness after treatment. The sensitivity analysis also showed that when wellness after treatment for chiropractic falls by 50 to 60 percent, PT would be the cost-effective option.

## 4. Discussion

Chiropractic care and PT are shown to be the superior non-pharmacologic strategies for treating LBP, when compared to other non-pharmacologic treatments [28]. However, studies have not been decisive on which one of these treatments is an optimal choice for acute LBP, as they both have been subject to much criticism. Many argued that adverse events after chiropractic treatment can be severe [29]. Specifically, they pointed at the adverse effects of X-ray imaging, which is essential for contemporary chiropractic. Studies showed that a low dose of X-rays may cause minor damages to biomolecules, while exposures above known threshold dose levels could result in harmful effects, including radiation illness [11,30]. It has also been shown that chiropractic therapy has cured only a marginal number of individuals in the sample [31]. Comparably, PT has shown to have some shortcomings in treating LBP. For example, a survey of physiotherapists showed that they believe their training has not instilled them with the requisite skills and confidence to successfully address and treat the multidimensional pain presentations seen in LBP [28].

This study analyzed these two strategies and showed that in the short term, chiropractic care is a more cost-effective alternative compared to PT for the treatment of acute low back pain. Chiropractic resulted in a lower cost ($48.56) and higher DALY (0.0043) than the PT over a one-month treatment period and five months follow-up. However, the marginal cost-effectiveness of chiropractic over PT suggests that both treatments were quite similar. Such findings are in line with the earlier studies, which found that the effectiveness and total costs of chiropractic and PT as primary treatments were similar to each other right after treatment and after 6 months follow-up [3,22,32]. 

This study has some limitations. First, this study did not consider the fraction of the subjects who underwent both chiropractic and PT, e.g., it only considered patients who only received chiropractic compared to those who only received PT. It is possible that by accounting for the received cross-therapy in the decision tree model, a larger contrast in terms of economic costs to patients and the DALY index would be yielded. Second, the negative risk of radiation in chiropractic was overlooked in this analysis. Taking imaging effect into consideration may attenuate the effectiveness of chiropractic therapy. Third, the small size of the study sample adds to the limitations of this research. Fourth, this study does not account for pregnant patients or those who might have other comorbidities [33]. Fifth, the difference in terms of economic costs and the DALY index alone may not indicate the benefits of using chiropractic therapy. Hence, it is difficult to defend any conclusions about the economic impact of chiropractic in patients presenting with LBP [34].

## Figures and Tables

**Table 1:** Baseline characteristics of patients.

Treatment Strategies ^1^	Ch	PT
Subjects	55%	45%
Mean age (SD)	41.4 (11.6)	40.5 (11.9)
Percent Women	60%	65%
Smokers	36%	31%
History of LBP	72%	70%
Previously treated for LBP	24%	35%
Duration of sick leave before treatment		
≤1wk	61%	58%
1–4 wk	37%	33%
Current episode of LBP		
≤6 wk	55%	48%
Using pain medication	20%	26%
Sick leave		
After treatment	40%	43%
At 6 mo	48%	46%
No of treatment sessions of Ch or Pt0-6 months	4.9	6.4

^1^ Ch represents Chiropractic, Pt represents physical Therapy.

**Table 2:** Detailed costs of treatment strategies for the years 1995 and 2018.

Treatment Strategies ^1^	1995 $		2018 $	
	Ch ^1^	PT ^1^	Ch	PT
Treatment	28.01	49.99	56.94	101.62
Imaging	67.26	-	136.73	-
Visit	114	134	231.74	272.39
Drug (Copay)	10	10	20.33	20.33

^1^ Ch represents Chiropractic, Pt represents Physical Therapy.

**Table 3:** The cost-effectiveness table.

	Ch^1^	PT^1^	Diff
Costs	$410.89	$459.45	$48.56
DALY	0.01562	0.01132	(0.0043)

^1^ Ch represents Chiropractic, Pt represents physical Therapy.
